# Brief Report: A Preference for Biological Motion Predicts a Reduction in Symptom Severity 1 Year Later in Preschoolers with Autism Spectrum Disorders

**DOI:** 10.3389/fpsyt.2016.00143

**Published:** 2016-08-24

**Authors:** Martina Franchini, Hilary Wood de Wilde, Bronwyn Glaser, Edouard Gentaz, Stephan Eliez, Marie Schaer

**Affiliations:** ^1^Office Médico-Pédagogique, University of Geneva, Geneva, Switzerland; ^2^Psychology and Educational Sciences, University of Geneva, Geneva, Switzerland; ^3^Department of Medical Genetics, Geneva University Medical School, Geneva, Switzerland; ^4^Stanford Cognitive & Systems Neuroscience Laboratory, Stanford University, Palo Alto, CA, USA

**Keywords:** autism spectrum disorders, early development, social orienting, eye-tracking, symptom severity, adaptive functioning

## Abstract

Recent research has consistently demonstrated reduced orienting to social stimuli in samples of young children with autism spectrum disorders (ASD). However, social orienting greatly varies between individual children on the spectrum. Better understanding this heterogeneity in social orienting may contribute to our comprehension of the mechanisms underlying autistic symptoms thereby improving our ability to intervene. Indeed, children on the autism spectrum who show higher levels of interest in social stimuli demonstrate reduced clinical symptoms and increased adaptive functioning. However, longitudinal studies examining the influence of social orienting on subsequent outcome are critically lacking. Here, we aim to explore the relationship between social interest at the age of 3 and changes in severity of autistic symptoms over the subsequent year, in 20 children with ASD and 20 age-matched typically developing (TD) children. A visual preference for social stimuli was measured using an eye-tracking task at baseline, consisting of a previously studied visual preference paradigm presenting biological and geometric motion side-by-side. The task was altered for the current study by alternating presentation side for each type of stimuli to keep visual perseveration from influencing participants’ first fixation location. Clinical data were collected both at baseline and 1 year later at follow-up. As a group, we observed reduced interest for biological motion (BIO-M) in children with ASD compared to TD children, corroborating previous findings. We also confirmed that a preference for BIO-M is associated with better adaptive functioning in preschoolers with ASD. Most importantly, our longitudinal results showed that a preference for BIO-M strongly predicted decreased severity of diagnostic symptoms. Participants who preferred social stimuli at the age of 3 showed drastic reductions in their severity level of autistic symptoms 1 year later, whereas participants who preferred geometric stimuli showed autistic symptoms that were unchanged or more severe after 1 year. As a whole, our results suggest that a preference for BIO-M may be key to understanding the behavioral phenotype of young children with ASD, and may represent a promising candidate behavior for predicting early developmental trajectories and outcome.

## Introduction

Autism spectrum disorders (ASD) comprise behavioral symptomatology with significant heterogeneity (1–3). This heterogeneity is evident from the first year of life, when behavioral signs of autism are first detectable (4). Also, developmental trajectories of young individuals with ASD have been shown to be varied and related on the behavioral features noted when the diagnosis of autism was established (5–7). For example, the rare, but consistent, longitudinal studies of outcome predictors, which are based on symptoms in children with ASD during early development, suggest that higher levels of early adaptive, cognitive and socio-communicative functioning predict better clinical outcomes [e.g., Ref. (5, 7)].

Several studies aimed at disentangling the heterogeneity of ASD symptoms have searched for biomarkers for autism with inconsistent results [for a review, see Ref. (8)]. Eye-tracking represents a promising way to measure early and specific visual exploration patterns in ASD that are different from those of children with typical development or developmental delay [for a review, see Ref. (9)]. Most eye-tracking studies on young children with autism have used stimuli with a high social content, such as faces or biological motion (BIO-M) (human movement) [e.g., Ref. (10–12)]. These investigations using social stimuli have consistently reported reduced attention to social stimuli in individuals with ASD [for a meta-analysis see Ref. (13)], though this reduction is inconsistent in preschoolers with ASD and appears to be related to adaptive or cognitive functioning [e.g., Ref. (14–17)]. These initial findings provide evidence for a relationship between reduced social interest in young children on the spectrum and their development. However, it is important to keep in mind that paradigm design also can greatly influence attention to social stimuli. An elegant meta-analysis by Chita-Tegmark (13) recently described different eye-tracking paradigms used to assess visual attention to social stimuli in ASD. This implies, for instance, social scenes used to measure preference for socially salient stimuli (e.g., faces) or on non-socially salient stimuli (e.g., objects). Also, existing research used visual preference paradigms opposing social stimuli to non-social stimuli (e.g., BIO-M vs. non-BIO-M). Measures of preference are both considered as visual exploration (e.g., time spent on stimuli) or latency to fixate social stimuli (e.g., reaction time). The authors point out that non-central aspects of these paradigms, including motion (e.g., static vs. dynamic stimuli), social content and communicative intention (e.g., number of people in a social scene and their social engagement), ecological validity, and multimodal presentation (e.g., conjunction with audio inputs) can significantly affect the allocation of social attention.

The *social motivation hypothesis of autism* [for a review, see Ref. (18)] considers a lack of social interest as the trigger for a behavioral cascade leading to the emergence of ASD symptomatology (19, 20). Accordingly, studies that further explore the relationship between reduced social interest and autistic symptoms deepen our understanding of early development of ASD in individuals. However, to the best of our knowledge, researchers have yet to explore the relationship between social interest and the development of severity of diagnostic symptoms in preschoolers with ASD over time.

Here, we explore how reduced social interest impacts ASD symptomatology in a group of 20 preschoolers with ASD and in 20 typically developing (TD) children of the same age. Pierce and colleagues (10, 16) previously demonstrated the validity of using visual preference patterns for BIO-M to assess social interest in preschoolers with ASD. Their task inspired our eye-tracking paradigm of visual preference for BIO-M (Franchini et al., submitted)^1^, redesigned for the current study. In addition to the eye-tracking paradigm, we also evaluated participants on severity of diagnostic symptoms during a diagnostic assessment and adaptive functioning using parent reports to investigate whether visual preference, clinical, and/or adaptive features predict severity of diagnostic symptoms 1 year later.

First, we would expect that preschoolers with ASD would spend a lower percentage of their time looking at BIO-M than TD preschoolers; second, that the time ASD and TD participants spend on the BIO-M would correlate with their clinical and adaptive functioning. Third, we hypothesize that a preference for BIO-M will be positively related to a reduction in severity of diagnostic symptoms after 1 year.

## Materials and Methods

A total of 20 participants with ASD (all boys) were included in the study. A first visit (Time 1) was planned as soon as possible after a clinical diagnosis was made and a follow-up visit (Time 2) was organized 12 months later. An age-matched group (all boys) of TD children was compared to our group of children with ASD at Time 1 only. Participants with ASD were recruited through French-speaking parent associations and specialized clinical centers. TD participants were recruited through announcements in the Geneva community. At Time 1, the children’s ages ranged between 22 and 51 months. The groups did not differ by age (*t* = 0.38, *p* = 0.71, see Table 1). At Time 2, ASD participants were between 33 and 63 months of age (mean age = 47.13, SD = 9.54). As expected, the children in the ASD group and the TD groups differed on measures of severity of symptoms and adaptive behavior (further details in Table 1). The Institutional Review Board of the University of Geneva approved the study protocol for all participants, and participants’ parents gave their informed consent prior to inclusion in the study.

**Table 1:** **Comparison between ASD and TD children on their clinical, adaptive, and social orienting features at Time 1**.

	ASD – mean (SD) *n* = 20, males	TD – mean (SD) *n* = 20, males	*t*-Value	*p*-Value
Age (in months)	35.00 (9.47)	33.84 (9.52)	0.38	0.71
Severity Score	6.80 (0.39)	1.05 (0.05)	14.31	<0.0001***
Adaptive Behavior Composite	73.15 (1.87)	102.6 (2.78)	8.87	<0.0001***
Communication	73.00 (2.08)	104.8 (2.28)	9.86	<0.0001***
Daily Living Skills	75.90 (2.45)	102.4 (2.43)	7.68	<0.0001***
Socialization	74.45 (1.59)	99.84 (3.16)	7.58	<0.0001***
Motor Skills	83.37 (2.64)	98.95 (2.57)	4.23	0.0002***
Preference for BIO-M (%)	44.83 (4.98)	68.30 (2.29)	4.21	0.0002***

***p < 0.001.

### Measures

To measure adaptive behavior, we used the Vineland Adaptive Behavior Scales, 2nd edition (21). The VABS-II is a standardized parent report interview that provides a standardized Adaptive Behavior Composite and four domain scores: Communication, Daily Living Skills, Socialization, and Motor Skills.

To assess the severity of autistic symptoms, we used the Autism Diagnostic Observation Schedule – Generic (ADOS-G) (22), which gives a diagnostic score that is derived from the sum of each symptom score according to diagnosis of ASD in the DSM-5 (23). The ADOS-G diagnostic score was then transformed according to Gotham et al.’s algorithm (24) to obtain a Severity Score ranging from 1 to 10. Severity Scores allow further comparison of the severity of autism spectrum symptoms between the ADOS-G evaluations of each participant. All participants performed the Module 1 of the ADOS-G (module adapted to children with “Few to No Words” or “Some Words”) or Module 2 (adapted to children with “Phrase Speech”).

Finally, to quantify participants’ preference for BIO-M, we designed a Biological Motion Visual Preference eye-tracking task (BVMP, see text footnote 1), inspired from the paradigm proposed by Pierce et al. (10). This passive 1-min task consists of the simultaneous presentation of dynamic geometric motion (GEO-M) on one side of the eye-tracking screen, and dynamic BIO-M on the other side. For the GEO-M, we used moving geometric shapes, similar to the classic abstract screen savers taken from MacOS screensavers or available under General Public License.^2^ For the BIO-M, we recorded standardized sequences of one child moving and dancing in front of a white wall (to minimize opportunities for distraction). In contrast to the stationary sequence of 60 s used by Pierce et al. (10), the stimuli in our study alternated between the left and right sides. Between each segment, a turning wheel brought the child’s gaze to the middle of the screen. Of the six segments of children moving and dancing, three sequences were of a boy and three were of a girl.

The task was administered using Tobii Studio software^3^ with the TX300 Tobii eye-tracker. The sampling rate of the machine was 300 Hz and the video resolution was 1920 × 1080 pixels. As recommended by Tobii, participants sat on a parent’s lap or alone on a chair at approximately 60 cm from the screen to minimize the impact of head movement on the gaze data. Before administering the task, all participants completed a five-point calibration procedure adapted to toddlers to detect eye motion and eye gaze.

Data analysis was done with Tobii Studio, version 3.1.6. The software automatically counts a fixation point every time a participant spends at least 100 ms within a 30-pixel circle. Areas of interests (AOIs) were drawn on the paradigm videos to delineate BIO-M and GEO-M. For each kind of stimuli (BIO-M and GEO-M), we calculated the total sum of fixation duration (the total looking time). The percentage of time spent on each stimulus type (BIO-M and GEO-M) was calculated by dividing the total fixations on in each AOI (or per stimulus type) by the total fixations on the entire screen. Visual preference for BIO-M was defined by whether a child looked at least 50% of the time at the video of a child moving and dancing.

### Analysis Strategy

We first performed descriptive analyses of the groups’ behavior and scores at Time 1. Children with ASD and TD children were compared on time spent on BIO-M at Time 1 by means of a *t*-test. To explore the relationship between variables in the ASD and the TD groups at Time 1, we correlated preference for BIO-M with standard VABS-II domain scores. In the ASD group, we additionally correlated Severity Score from the ADOS-G with VABS-II domain scores and with a preference for BIO-M (this was not performed in the TD group because all but one TD participant received the lowest possible Severity Score of 1). Subsequently, according to previous work [Pierce and colleagues (10, 16) and see text footnote 1], we split our group of children with ASD into two sub-groups: Social Responders (SR-ASD, who spent more than 50% of their total time on BIO-M) and Geometric Responders (GR-ASD, who spent more than 50% of their total time on GEO-M). Using *t*-tests, we compared GR-ASD and SR-ASD groups on the VABS-II domain scores and on the Severity Score from the ADOS-G.

Second, in the ASD group only, we correlated VABS-II scores and percent time spent on BIO-M at Time 1 with percent of change in Severity Score between Time 1 and Time 2. Correlation analyses were performed using Pearson correlation coefficients. Using a *t*-test, we then compared GR-ASD and SR-ASD children on their percent of change in Severity Score between Time 1 and Time 2. We then observed GR-ASD and SR-ASD improvements on each ADOS-G item contributing to the Severity Score. We, therefore, separated GR-ASD and SR-ASD into three groups for chi-square analysis of each item in the Severity Score: participants who showed a reduction on the item, participants who demonstrated no change on the item and participants with increased severity on the item. Three items were not included in our analyses because they contributed to the Severity Score in either Module 1-Few to No Words or Module 1-Some Words and, thus, did not allow for sufficient data. The specific items were “Pointing” (*n* = 7), “Responding to Joint Attention” (*n* = 4), and “Intonation of Vocalizations or Verbalizations” (*n* = 4). Results were considered significant at a *p*-value of 0.05. For the correlations between the VABS-II domains and related variables (preference for BIO-M, Severity Score, and percent change in Severity Score), we applied a Benjamini–Hochberg correction for multiple comparisons.

## Results

### Cross-Sectional Observations: A Reduced Preference for Biological Motion in ASD Preschoolers Compared to TD Preschoolers

The ASD and TD groups differed on time spent on BIO-M (*t* = 4.21, *p* < 0.001) during the BVMP task (see Table 1). All TD children showed a preference for BIO-M (they spent more than 50% of the total time on BIO-M). By contrast, in the group of children with ASD, 12 preferred GEO-M (thus comprising the GR-ASD sub-group) and 8 preferred BIO-M (comprising the SR-ASD sub-group).

Time spent on BIO-M correlated with the VABS-II Adaptive Behavior Composite scores in the group of children with ASD (*r* = 0.75, *p* < 0.001, *df* = 18) but not in the TD group (*r* = 0.45, 0.05, *df* = 18) in which we observed a trend toward significance. We then examined the relationship between a preference for BIO-M and VABS-II domain scores in both groups.

After applying a Benjamini–Hochberg correction (*p* < 0.0125), we observed a positive relationship in the ASD group between time spent on BIO-M and scores on the Daily Living Skills (*r* = 0.55, *p* = 0.0117, *df* = 18) and the Socialization (*r* = 0.84, *p* < 0.001, *df* = 18) domains. We observed a non-significant trend between time spent on BIO-M and scores on the Motor Skills (*r* = 0.51, *p* = 0.02, *df* = 18) and Communication (*r* = 0.68, *p* = 0.047, *df* = 18) domains. In the TD group, we did not observe any significant relationship between preference for BIO-M and scores on the Communication (*r* = 0.29, *p* = 0.22, *df* = 18), Daily Living Skills (*r* = 0.03, *p* = 0.89, *df* = 18), Socialization (*r* = 0.16, *p* = 0.51, *df* = 18) or Motor Skills (*r* = 0.51, *p* = 0.03, *df* = 18) domains, though we observed a trend toward significance in the latter.

When we correlated severity of symptoms and VABS-II scores in the ASD group, we observed a trend toward a negative correlation between Severity Score and the Adaptive Behavior Composite (*r* = −0.40, *p* = 0.08, *df* = 18). Correcting the *p*-values for multiple comparisons at the threshold of *p* < 0.0125, the correlation between the Severity Score and the VABS-II socialization score did not survive (*r* = −0.44; *p* = 0.05, *df* = 18); none of the other correlations survived either between the Severity Score and the VABS-II Communication (*r* = −0.29, *p* = 0.22, *df* = 18), Motor Skills (*r* = −0.38, *p* = 0.11, *df* = 18) or Daily Living Skills (*r* = −0.41, *p* = 0.07, *df* = 18) scores. Time spent on BIO-M in the ASD group was not correlated with Symptom Severity (*r* = −0.22, *p* = 0.36, *df* = 18).

We subsequently compared SR-ASD and GR-ASD children. Despite the small sample sizes, we observed a significant difference between the groups. The SR-ASD children showed higher adaptive functioning compared to GR-ASD for the Adaptive Behavior Composite, Communication, and Socialization domains (*t* = 2.13, *p* = 0.047; *t* = 2.14, *p* = 0.047; *t* = 3.39, *p* < 0.01), but not for the Daily Living Skills and Motor Skills domains (*t* = 1.17, *p* = 0.26; *t* = 1.01, *p* = 0.33). We did not, however, observe a significant difference between SR-ASD and GR-ASD children (*t* = 0.67, *p* = 0.51) on Severity Score.

### Longitudinal Analyses: A Preference for Biological Motion Predicts Reduced Symptom Severity 1 Year Later

**Figure 1:**
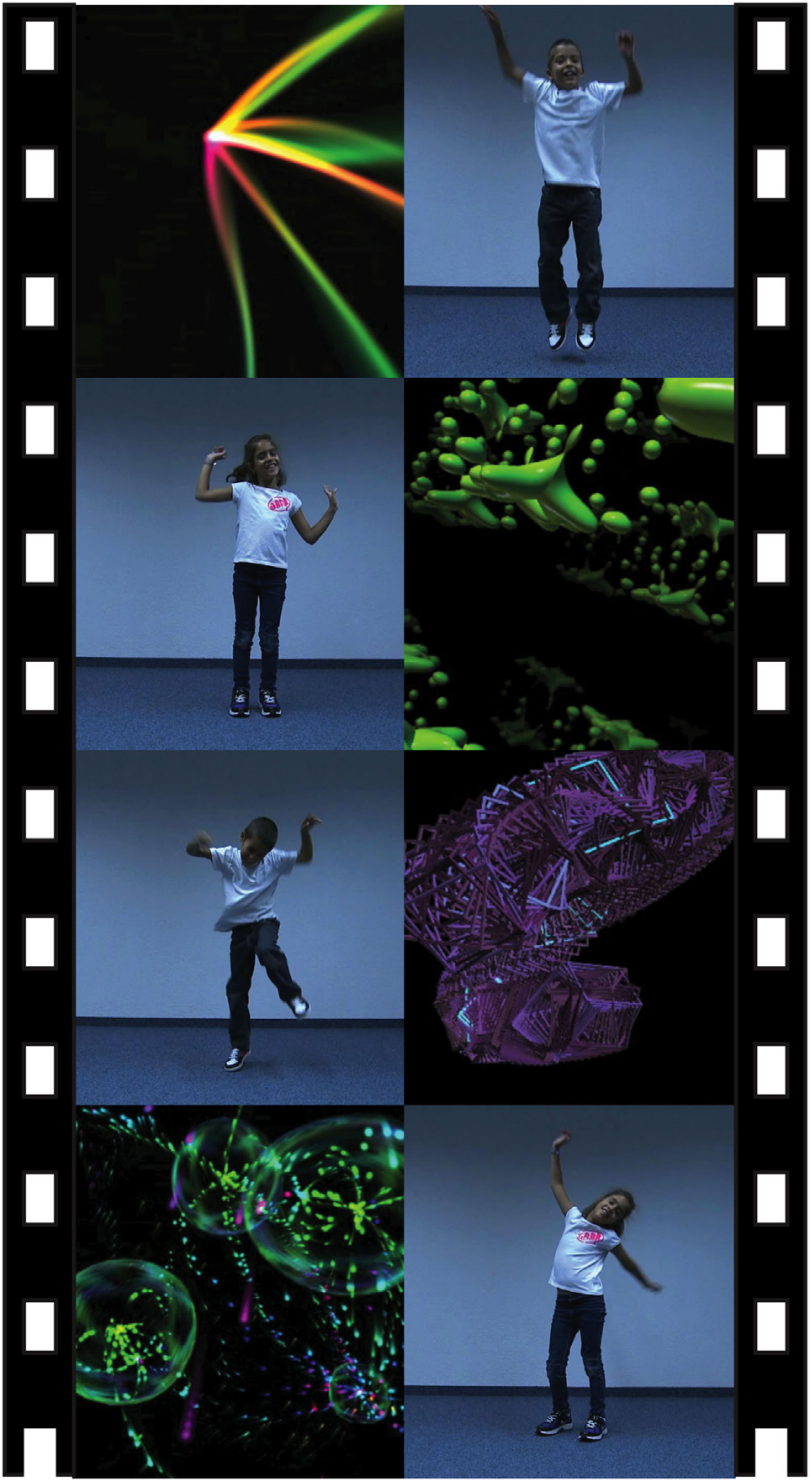
**Screenshots representing examples from the BMVP task**.

We first correlated time spent on BIO-M at T1 and VABS-II adaptive behavior scores at T1 with percent change (between T1 and T2) in Severity Score on the ADOS-G (results are shown in Table 2). The results suggest that time spent on BIO-M at T1 predicts reduction in Severity Score 1 year later in the ASD group (*r* = −0.67, *p* = 0.001, *df* = 18, see Figure 1). In other words, children who spent the majority of their time on BIO-M during the eye-tracking BMVP task at baseline were the individuals who showed the most clinical improvement, as measured by the Symptom Severity score 1 year later.

**Table 2:** **Predictors of changes in symptom severity 1 year later**.

Adaptive behavior or preference for BIO-M at Time 1	Reduction in Severity Score from Time 1 to Time 2	
	*r*	*p*-value
Adaptive Behavior Composite	−0.31	0.18
Communication	−0.47	0.037
Daily Living Skills	−0.07	0.78
Socialization	−0.31	0.19
Motor Skills	−0.10	0.66
Preference for BIO-M (%)	−0.67	0.001**

**p < 0.01.

**Figure 2:**
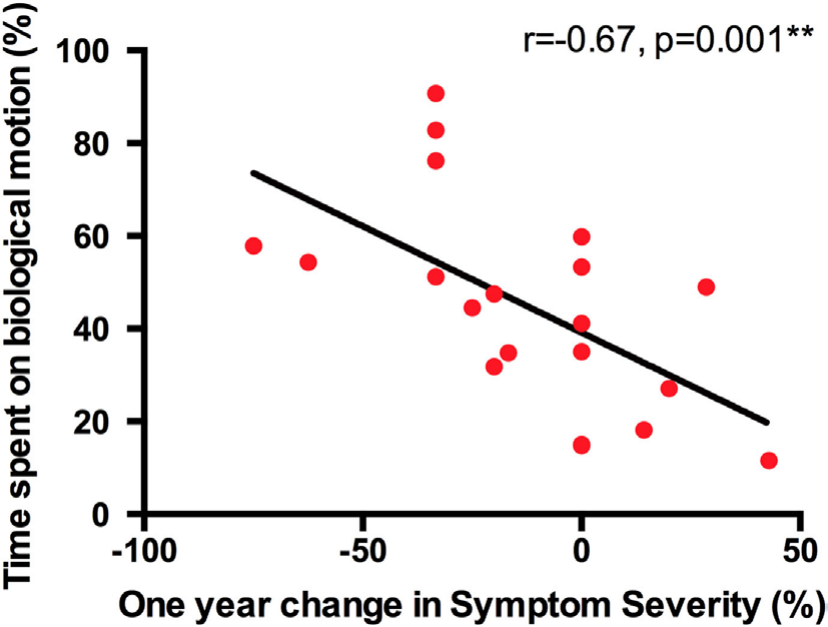
**Time spent on biological motion strongly predicts 1-year reduction of Symptom Severity in the group of preschoolers with ASD**.

A correlation between VABS-II Communication score and percent change in Severity Score survived the uncorrected significance threshold, but did not survive our correction for multiple comparisons (*r* = −0.47, *p* = 0.037, *df* = 18).

We then looked at differences between the SR-ASD and GR-ASD sub-groups on the change in Symptom Severity between Time 1 and Time 2, and found a group difference. SR-ASD children showed greater improvement in Symptom Severity than GR-ASD children (*t* = 3.38, *p* = 0.003). After that, we looked at inter-group differences between GR-ASD and SR-ASD children for each item that contributed to the Symptom Severity score. Two participants were administered a Module 2 ADOS-G at one or both of the two visits and were, thus, excluded from analysis of items “Frequency of Spontaneous Vocalization Directed to Others” and “Integration of Gaze and Other Behaviors during Social Overtures,” items that count toward the Severity Score in Module 1 only. A significant difference, with SR-ASD children showing more improvement than GR-ASD children, was detected on the following items: “Shared Enjoyment in Interaction” (*X*^2^ = 7.78, *p* = 0.02), “Showing” (*X*^2^ = 13.13, *p* = 0.001), and “Spontaneous Initiation of Joint Attention” (*X*^2^ = 7.82, *p* = 0.02). For details, see Table 3.

**Table 3:** **Change in ASD Symptom Severity after 1 year according to preference for BIO-M at Time 1**.

		Change in Symptom Severity				
ADOS-G Symptoms	Preference for BIO-M	Reduction (*n*)	No change (*n*)	Increasing (*n*)	*X*^2^	*p*-Value
**SOCIAL AFFECT**						
**Communication**						
Frequency of Spontaneous Vocalization Directed to Others	SR-ASD (*n* = 6)	5	1	0	4.06	0.13
	GR-ASD (*n* = 12)	4	7	1		
Gestures	SR-ASD (*n* = 8)	5	2	1	1.25	0.54
	GR-ASD (*n* = 12)	5	6	1		
**Reciprocal social interaction**
Unusual Eye Contact	SR-ASD (*n* = 8)	1	7	0	0.06	0.79
	GR-ASD (*n* = 12)	2	10	0		
Facial Expression Directed to Others	SR-ASD (*n* = 8)	2	6	0	0.06	0.79
	GR-ASD (*n* = 12)	3	6	3		
Integration of Gaze and Other Behaviors during Social Overtures	SR-ASD (*n* = 6)	4	2	0	2.00	0.38
	GR-ASD (*n* = 12)	5	4	3		
Shared Enjoyment in Interaction	SR-ASD (*n* = 8)	5	3	0	7.78	0.02*
	GR-ASD (*n* = 12)	1	7	4		
Showing	SR-ASD (*n* = 8)	6	1	1	13.13	0.001**
	GR-ASD (*n* = 12)	0	9	3		
Spontaneous Initiation of Joint Attention	SR-ASD (*n* = 8)	5	1	2	7.82	0.02*
	GR-ASD (*n* = 12)	1	8	3		
Quality of Social Overtures	SR-ASD (*n* = 8)	3	5	0	2.55	0.28
	GR-ASD (*n* = 12)	1	11	0		
**RESTRICTED AND REPETITIVE BEHAVIOR**						
Unusual Sensory Interest in Play Material/Person	SR-ASD (*n* = 8)	2	6	0	3.34	0.16
	GR-ASD (*n* = 12)	3	5	4		
Hand and Fingers and Other Complex Mannerisms	SR-ASD (*n* = 8)	2	6	0	4.47	0.11
	GR-ASD (*n* = 12)	2	5	5		
Unusually Repetitive Interests or Stereotyped Behavior	SR-ASD (*n* = 8)	4	2	2	3.61	0.16
	GR-ASD (*n* = 12)	2	8	2		

*p < 0.05, **p < 0.01.

## Discussion

Our results showing reduced interest in BIO-M in preschoolers with ASD compared to TD children, corroborate previous results by Pierce and colleagues (10, 16) and by our research group (see text footnote 1). Our ASD group also demonstrated a wide variance in their preferences for BIO-M, with percentage of visual preference for BIO-M ranging from 11.6 to 90.17% between participants. As previously observed in children with autism [(10, 16), see text footnote 1], this heterogeneity was related to their adaptive functioning. Based on these cross-sectional observations, we subsequently explored whether measures of adaptive functioning and social interest could predict symptom development in preschoolers on the spectrum. Longitudinal analyses revealed that a child’s preference for BIO-M predicted a reduction in the severity of symptomatology after 1 year. Moreover, children with ASD with a stronger preference for BIO-M (SR-ASD) showed improvement on “Shared Enjoyment in Interaction,” “Showing,” and “Spontaneous Initiation of Joint Attention.”

The implications of reduced social interest overall during the early development of children with ASD and the relationship between social interest (a preference for BIO-M) and reductions in certain ASD symptoms will be discussed in the following sections.

### Reduced Preference for Biological Motion in ASD Children Compared to Their TD Children

In the present study, we confirmed previous findings (10, 16) that preschoolers with ASD orient less often toward BIO-M than TD children. As discussed in Franchini et al. (see text footnote 1), these results demonstrate that visual preference paradigms for BIO-M can serve as consistent and valid biomarkers for visual exploration patterns in young children with ASD. Moreover, presentation side of BIO-M and GEO-M stimuli changed often during the BMVP task to avoid visual exploration patterns associated with “sticky attention,” which are characterized by a difficulty disengaging from GEO-M, a behavior that may be characteristic of individuals on the autism spectrum (25). Pierce and collaborators (10, 16) did not alternate the stimuli presentation during their task, which could potentially result in the young participants’ attention to get “stuck.” Accordingly, social orienting may, thus, be a better indicator of an initial preference for social or geometric stimuli, rather than a participant’s main interest during the task. As we hypothesized, a preference for BIO-M was related to adaptive functioning in participants on the autism spectrum, lending support to the idea that interest in social stimuli is related to the behavioral phenotype of young children with ASD.

It is important to note that there are studies on social attention in ASD that did not report a difference between the visual preferences of ASD and TD children for social stimuli [for reviews, see Ref. (13, 26)]. Specific social content of stimuli used in tasks, as well as the salience of non-social stimuli can influence visual attention patterns in individuals with ASD [for a review, see Ref. (26)] and contribute to differences between studies. However, as Chita-Tegmark suggested in a review (13), tasks that display moving biological and geometric stimuli on opposite sides of the display, such as the task used in the current study and the one used by Pierce et al. (10, 16), have the advantage of clearly demarcating a visual preference for BIO-M or GEO-M in children with ASD.

### A Preference for Biological Motion Predicts a Reduction in Symptom Severity 1 Year Later

To date, only a few research studies using limited samples have explored the evolution of Symptom Severity in young children with ASD. One such study demonstrated the idea that superior adaptive behavior is associated with better outcome in preschoolers with ASD (5). Also, better adaptive functioning has indeed been shown to be predictive of a small subset of young children who show “optimal progress” between ages 2 and 4, including those who no longer meet criteria for diagnosis at age 4 (7). In our study, we found that the best predictor of clinical improvement in Symptom Severity was a preference for BIO-M. Despite our small sample size, our results suggest that a preference for BIO-M may be a key to predicting early developmental trajectories and outcome.

Finally, our results point to specific criteria within the diagnostic assessment (ADOS-G) that were most improved in children with a preference for BIO-M. These items are “Shared Enjoyment in Interaction,” “Showing,” and “Spontaneous Initiation of Joint Attention,” Interestingly, these three items account for the “Social Affect” domain of the ADOS-G. According to a recent publication by Mundy et al. (27), these three criteria measure the initiation of joint attention behaviors, which also are described as “*infants’ use of gestures and eye contact to direct others’ attention to objects, to events, and to themselves*” [(28), p. 269]. The initiation of joint attention in early development of children with ASD is an example of a spontaneous and natural attention-sharing behavior that is intrinsically related to both social attention and social learning [for a review, see Ref. (28)]. Accordingly, increased social interest, as measured by a preference for BIO-M in the current study, may be related to early improvements in joint attention behaviors, thus resulting in improved early socio-communicative development (19, 20). These results corroborate the *social motivation hypothesis of autism* [for a review, see Ref. (18)], in that a preference for early social interest appears to support ensuing socio-communicative learning in young children with autism.

### Limitations and Perspectives

The present study has several limitations. First, our sample size was relatively small. However, despite our small sample size, our results demonstrate that clinical differences can be detected during a 60-s standardized video, a quick and easy screening idea that warrants replication and further exploration. Second, this study explores clinical outcome of children with ASD during a 1-year period only. While future investigations should try to explore the influence of early social interest over a longer developmental period and with more participants, we are convinced that the strength of our statistical results reflects the importance of social orienting in ASD during preschool, a period that appears to be particularly ripe for developmental changes. Finally, subsequent studies may wish to explore the relationship between social interest and Symptom Severity taking into account baseline behavioral features of children with ASD, as well as specific therapeutic interventions, to better understand the factors moderating the longitudinal change.

## Conclusion

Social attention plays an important role in early life, and may be a pivotal factor for individuals on the autism spectrum. Measures of social interest, as the one described in this study, offer the potential to better understand clinical features and outcome during early development in children with ASD. Our results provide support for this idea, by demonstrating a way of operationalizing social interest in young children to better understand and follow the heterogeneous phenotype between individuals on the spectrum. The resulting data are especially useful for the conceptualization of individualized early intervention in ASD and the assessment of progress during intervention.

## Ethics Statement

The local ethical commission of Geneva approved this study.

## Author Contributions

MF, HW, BG, SE, and MS designed the study; MF, HW, and MS acquired the data; MF and MS analyzed the data and wrote the first draft of the article; MF, HW, BG, EG, SE, and MS contributed to the interpretation of the results and the writing of the manuscript. All authors have approved the final manuscript.

## Conflict of Interest Statement

The authors declare that the research was conducted in the absence of any commercial or financial relationships that could be construed as a potential conflict of interest.

## Footnotes

^1^Franchini M, Wood de Wilde H, Glaser B, Gentaz E, Eliez S, Schaer M. Social orienting and joint attention in preschoolers with Autism Spectrum Disorders. Submitted.

^2^http://www.reallyslick.com/screensavers

^3^www.tobii.com

## Abbreviations

ASD, autism spectrum disorders; BIO-M, biological motion; BVMP, biological motion visual preference task; GEO-M, geometric motion; GR, geometric responders; SR, social responders; TD, typically developing.
