# Corrigendum: Brief Report: A Preference for Biological Motion Predicts a Reduction in Symptom Severity 1 Year Later in Preschoolers with Autism Spectrum Disorders

**DOI:** 10.3389/fpsyt.2017.00058

**Published:** 2017-04-12

**Authors:** Martina Franchini, Hillary Wood de Wilde, Bronwyn Glaser, Edouard Gentaz, Stephan Eliez, Marie Schaer

**Affiliations:** ^1^Office Médico-Pédagogique, Geneva University, Geneva, Switzerland; ^2^Psychology and Educational Sciences, Geneva University, Geneva, Switzerland; ^3^Department of Medical Genetic, Geneva University Medical School, Geneva, Switzerland; ^4^Stanford Cognitive and Systems Neuroscience Laboratory, Stanford University, Palo Alto, CA, USA

**Keywords:** autism spectrum disorders, early development, social orienting, eye-tracking, symptom severity, adaptive functioning

**Text Correction**

In the original article, there was an error on the details about the filter that we used during our analyses: [The software automatically counts a fixation point every time a participant spends at least 100 ms within a 30-pixel circle.].

A correction has been made to **[Measures]**, **[Paragraph Number 5]**. Details about the filter that we used during analyses have been correctly stated:

[A I-VT filter was enabled during analysis. (Classifier: 30°/s; Velocity calculator window length: 20 ms). The merge fixations option was further enabled (Max. time between fixations: 75 ms; Max angle between fixations: 0.5°).]

The authors apologize for this error and state that this does not change the scientific conclusions of the article in any way.

## Conflict of Interest Statement

The authors declare that the research was conducted in the absence of any commercial or financial relationships that could be construed as a potential conflict of interest.

